# Adult Height in Girls With Idiopathic Premature Adrenarche: A Cohort Study and Design of a Predictive Model

**DOI:** 10.3389/fendo.2022.852422

**Published:** 2022-03-03

**Authors:** Francisco Javier Mejorado-Molano, María Luisa Sanz-Calvo, Ana Posada-Ayala, Nieves Caballo-Roig, Teresa Gavela-Pérez, Ignacio Mahillo-Fernández, Leandro Soriano-Guillén

**Affiliations:** ^1^ Department of Pediatrics, Instituto de Investigación Sanitaria (IIS)-Fundación Jiménez Díaz, Universidad Autónoma de Madrid, Madrid, Spain; ^2^ Centro de Salud Palma Norte, Madrid, Spain; ^3^ Centro de Salud Embajadores, Madrid, Spain; ^4^ Epidemiological Research Unit, Instituto de Investigación Sanitaria (IIS)-Fundación Jiménez Díaz, Madrid, Spain

**Keywords:** idiopathic premature adrenarche, adult height, target height, adult height predictive models, multiple regression analysis

## Abstract

**Introduction:**

Idiopathic premature adrenarche (IPA) is considered a normal variant of puberty, presenting more commonly in female patients. There are concerns as to whether IPA alters the final height of these girls. Our main objectives were to (a) compare the adult height of girls with IPA against their target height and (b) design a mathematical model to predict adult height at diagnosis in female patients with IPA.

**Materials and Methods:**

A cohort study of girls with IPA was conducted from the time of IPA diagnosis until adult height. The following data were collected: target height, perinatal history, anthropometric and biochemical variables and bone age at diagnosis, age at Tanner stage 2 and menarcheal age, and adult height. First, we performed a univariate statistical analysis after which we carried out a multiple linear regression analysis using adult height as the dependent variable.

**Results:**

We obtained data from 79 female patients diagnosed with IPA with a mean adult height of 164.6 cm (95% CI: 163.36–165.85 cm). The mean follow-up time was 6.60 years. Average age at Tanner stage 2 was 9.71 years. Mean menarcheal age was 11.64 years. There were no significant differences between target height and adult height. Of the several predictive models designed for these patients, one of them, which included bone age, obtained an *R* ^2^ value of 71%.

**Conclusions:**

Although slightly advanced puberty was observed among the girls with IPA, their adult height was preserved. The use of predictive models of adult height on diagnosis of IPA could facilitate closer follow-up of girls at risk of reduced adult height.

## Introduction

As the zona reticularis develops in the adrenal cortex, at approximately 5 to 8 years of age, there is a simultaneous increase of androgen precursors with weak androgenic activity, such as dehydroepiandrosterone (DHEA), its sulfate (DHEA-S), and androstenedione. This biological process, called adrenarche, is typical of humans and higher primates (1–3). Classically, a DHEA-S level higher than 40 µg/dl (1 µmol/l) has been considered a biochemical hallmark of adrenarche (4). Clinical signs of adrenarche can include presence of pubic and axillary hair, acne, greasy hair, and apocrine adult-type body odor (4, 5).

In girls, premature adrenarche (PA) is determined by the presence of clinical signs of androgen action before the age of 8 years in addition to androgen precursors that are abnormally high for the patient’s prepubertal stage (Tanner 1) and more suitable for more advanced Tanner stages (Tanner 2-4) (2). Once other causes of excess androgen production have been ruled out, such as adrenal or ovarian tumors, non-classical congenital adrenal hyperplasia, or exogenous androgen administration, a diagnosis of idiopathic premature adrenarche (IPA) may be considered, as IPA is a diagnosis of exclusion (6, 7).

PA is a relatively frequent reason for pediatric endocrinology consultations. However, epidemiological data are scarce. An elegant Finnish study reported an 8.6% prevalence of PA as defined by DHEA-S ≥ 1 µmol/L and presence of any clinical sign of androgen action in girls under age 8 years as compared to a rate of 1.8% in boys under age 9 years (8). Subsequent studies have provided further evidence that this entity is more common among girls (9).

Prepubertal girls with IPA are usually taller and have advanced bone age when compared to healthy prepubertal girls of the same age. Later, girls with IPA usually have more advanced pubertal development and earlier menarche (10). These findings raise concerns regarding the potential effect of this entity on final adult height. To date, however, there are few longitudinal studies evaluating adult height in girls with IPA (11).

The uncertainty related to adult height (11) together with the possibility of developing obesity, metabolic disturbances, and polycystic ovary syndrome at later ages (12, 13) creates a need for periodic follow-up of these patients. Though different models for predicting adult height have been proposed for other pubertal disorders such as central precocious puberty (14–16), to our knowledge, no previous publications have designed predictive models of adult height in female IPA that could aid in the follow-up of these patients. For this reason, we pursued a dual objective in this study: (a) to compare the adult height of a cohort of girls with IPA against their target height and with height-prediction tables for our country and (b) to design a model to predict adult height at IPA diagnosis.

## Materials and Methods

### Study Design

A cohort study was carried out including girls newly diagnosed with IPA from January 2007 until December 2015. Subsequently, the patients have been periodically evaluated in the Pediatric Endocrinology Unit of the Hospital Universitario Fundación Jiménez Díaz until adult height.

### Inclusion Criteria

We included girls with no underlying chronic disease or genetic disorders, who were not undergoing any medical treatment and met the diagnostic criteria for IPA (17), which were as follows:

✓ Appearance of pubic and/or axillary hair before age 8 years;
✓ Absence of thelarche according to Tanner stage 1 of breast development (18);
✓ DHEA-S > 40 µg/dl, though this was within the range of normal values for the Tanner stage of pubic hair development;
✓ Normal basal and stimulated 17-OH-progesterone values after ACTH stimulation test to rule out presence of non-classical congenital adrenal hyperplasia; and
✓ Normal abdominal and pelvic ultrasound to rule out presence of adrenal or ovarian tumor.

### Study Variables

- *Family data*: height of both parents (cm) was measured in the Pediatric Endocrinology Unit. The target height was calculated as follows: father’s height + mother’s height/2 − 6.5 cm. Likewise, the SDS for target height was calculated according to Spanish reference values (19). Maternal menarcheal age was also collected.
- *Personal history*: ethnicity, country of birth, type of delivery, gestational age (weeks), birth weight (grams), and birth length (cm). We calculated birth weight and birth length SDS using Spanish reference values according to gestational age and sex (20).
- *At diagnosis:* chronological age (years) and age of pubarche/axilarche onset (years). Height (cm), weight (kg), and body mass index (BMI) [weight (kg)/height (m^2^)] were also recorded. We calculated height, weight, and BMI SDS using references tables for the Spanish population (19). Bone age (BA) was always assessed in accordance with the Greulich & Pyle method (21) by the most trained physician of the Pediatric Endocrinologist Unit (LS-G). Predicted adult height was also calculated using the Bayley–Pinneau method (average age) (22) in cm and SDS (19). Pelvic and abdominal ultrasound were performed. Twelve-hour fasting blood samples were used to measure the hormonal parameters androstenedione (ng/ml), testosterone (ng/ml), DHEA-S (µg/dl), as well as baseline, 30’, and 60’ 17-OH-progesterone levels (ng/ml) after 250-µg ACTH stimulation test.
  ⚬ Androstenedione levels were determined by CLEIA (chemiluminescence enzyme immunoassay), the intra-assay coefficient of variation was 6.2%, and the inter-assay coefficient of variation was 8.5%. Testosterone concentrations were determined by ECLIA (electrochemiluminescence immunoassay), the intra-assay coefficient of variation was 1.6%, and the inter-assay coefficient of variation was 3.5%. DHEA-S concentrations were determined by ECLIA, the intra-assay coefficient of variation was 3.2%, and the inter-assay coefficient of variation was 4%. 17-OH-progesterone levels were determined by ELISA (enzyme-linked immunosorbent assay), the intra-assay coefficient of variation was 2.8%, and the inter-assay coefficient of variation was 5.8%.
- *Clinical follow-up:* every 6 months. We collected the following data on each visit: height (cm, SDS), weight (kg, SDS), BMI (kg/m^2^, SDS), growth velocity (cm, SDS), Tanner stage (breast development, pubic hair) (18), and menarcheal age.
- *Definitions:*
  ⚬ *Small for gestational age*: birth length and/or weight < −2 SDS (20).
  ⚬ *Obesity*: BMI > +2 SDS (19).
  ⚬ *Adult height:* height reached when the growth rate of the last year is <0.5 cm/year (calculated in cm and SDS).

### Subjects

A total of 79 girls with IPA were followed up until they reached adult height. Baseline characteristics are summarized in **Table 1**. At diagnosis, the mean chronological age was 7.63 years, while the mean BA was 8.49 years. In addition, the mean height at diagnosis was 1.56 SDS and the BMI was 0.71 SDS.

**Table 1:** Neonatal demographic and anthropometric data. Demographic, anthropometric, and biochemical data at diagnosis.

Variable (*n* = 79)	Mean (95% CI)
Gestational age (weeks)	39.14 (38.85–39.43)
Birth weight (SDS)	0.02 (−0.26–0.31)
Birth length (SDS)	0.31 (0.00–0.62)
Small for gestational age (*n*/%)	4/5
CA at diagnosis (years)	7.63 (7.35–7.90)
BA at diagnosis (years)	8.49 (8.14–8.83)
BA-CA (years)	0.85 (0.68–1.02)
Height (cm)	131.65 (129.43–133.87)
Height (SDS)	1.56 (1.36–1.77)
Adult height predicted by B&P (cm)	163.19 (161.78–164.60)
Adult height predicted by B&P (SDS)	0.34 (0.09–0.58)
BMI (SDS)	0.71 (0.42–1.00)
Obesity (*n*/%)	14/17.72
Testosterone (ng/ml)	0.15 (0.13–0.17)
Androstenedione (ng/ml)	0.72 (0.57–0.87)
DHEA-S (µg/dl)	79.56 (69.10–90.00)
Basal 17-OH-progesterone (ng/ml)	0.82 (0.68–0.96)
Peak 17-OH-progesterone after ACTH stimulation test (ng/ml)	3.30 (2.87–3.72)

CA, chronological age; BA, bone age; B&P, Bayley and Pinneau; BMI, body mass index.

### Ethics Approval

This observational study was performed in accordance with the principles of the Declaration of Helsinki, as well as the prevailing Spanish legislation on clinical research in human subjects. The study protocol was approved by the Research Ethics Committee of the University Hospital Fundación Jiménez Díaz (protocol n°: PIC003-19/Date: 30/01/2019). The purpose of the study was explained to all participants and both written parental consent and written assent from minors >12 years were obtained.

### Statistical Analyses

In the descriptive analysis, qualitative variables are shown in frequency tables and quantitative variables as mean and 95% confidence interval. The Kolmogorov–Smirnov test was applied to verify the normal distribution of the data. Those variables that did not follow a normal distribution were logarithmically transformed.

Student’s *t*-test for unpaired data was used to compare the adult height of girls with IPA with respect to target height.

Subsequently, the correlation between adult height and each of its potential predictive variables was evaluated, constructing a correlation matrix. Next, a multiple linear regression model was used, taking as the dependent variable adult height (cm) and as independent variables those that showed a significant relationship with adult height in the correlation matrix using a strategy of stepwise regression. Previously, the presence of collinearity between the independent variables included in the model was ruled out (correlation > 0.6). The best model was selected by obtaining the best adjusted coefficient of determination (*R* ^2^). The 95% confidence interval (CI) was also calculated for the predictions of these models.

The internal validation of the models was assessed by means of the leave-one-out cross validation (LOOCV) method (23). The validation process was summarized by the mean, standard deviation, and range of the absolute error. Loss of prediction was calculated as the difference between the multiple *R* ^2^ of the model fitted to the whole sample, and the *R* ^2^ of the validation process.

Associations or statistical differences with *p* values <0.05 were considered significant.

Analyses were performed using the SPSS version 25.0 software (IBM Corp., Armonk, NY, USA).

## Results

The mean age at diagnosis was 7.63 years, and the mean age at assessment of adult height was 14.16 years. Thus, the mean time of follow-up was 6.60 years. None of the girls was required to re-evaluate the BA during this follow-up.

Four girls out of a total of 79 met the criteria for small for gestational age (5%).

Tanner stage 2 of breast development was reached at the age of 9.71 years. In addition, mean menarcheal age was 11.64 years. Notably, maternal menarcheal age was 12.13 years.

The mean adult height of girls with IPA was 164.60 cm (163.36-165.85). When compared to their target height [163.56 cm (162.39–164.73)], no significant differences were observed (see **Table 2**). There were also no significant differences evidenced when the mean adult height was compared to the predicted adult height at diagnosis. None of the girls with IPA presented an adult height < −2 SDS. Moreover, 17.70% of girls had a difference of more than 1 SDS between their adult height and target height.

**Table 2:** Clinical follow-up of girls with IPA.

Variable (*n* = 79)	Mean (95% CI)	*p*-value
Growth velocity, first year (cm/year)	6.69 (6.39–7.00)	
Growth velocity, first year (SDS)	1.47 (1.08–1.85)	
Growth velocity, second year (cm/year)	6.68 (6.24–7.12)	
Growth velocity, second year (SDS)	1.70 (1.16–2.25)	
Tanner stage 2 breast development (years)	9.71 (9.49–9.94)	
Menarcheal age (years)	11.64 (11.39–11.89)	
Maternal menarcheal age (years)	12.13 (11.82–12.45)	
Maternal menarcheal age-menarcheal age (years)	0.52 (0.14–0.89)	0.008
Time of follow-up from diagnosis until adult height (years)	6.60 (6.12–7.09)	
Adult height (cm)	164.60 (163.36–165.85)	
Adult height (SDS)	0.58 (0.37–0.80)	
Growth from diagnosis until adult height (cm)	32.95 (31.09–34.82)	
Growth from Tanner stage 2 breast development until adult height (cm)	19.39 (18.26–20.52)	
Target height (cm)	163.56 (162.39–164.73)	
Target height (SDS)	0.40 (0.20–0.61)	
Adult height–Predicted adult height (cm)	1.71 (0.59–2.83)	0.003
Adult height–Target height (cm)	1.05 (-0.07–2.16)	0.066
BMI at adult height (SDS)	1.25 (0.67–1.84)	
BMI at adult height–BMI at diagnosis (SDS)	0.49 (0.07–0.90)	0.022
Obesity at adult height (*n*/%)	17/21.51	

BMI, body mass index.

**Table 3** summarizes different reports on adult height data in girls with a history of PA (11, 12, 24–27).

**Table 3:** Different studies of adult height-related data in girls with a history of PA.

Year (reference)	Country	*n*/type of study	Adult height*(cm)	Target height*(cm)	Adult height, control group* (cm)	Onset of puberty(years)	Mean menarcheal age(years)
2000 (24)	Italy	38/cohort	162.7 (160.8–164.6)	159.5 (157.7–161.3)	–	9.9	12.2
2006 (25)	Spain	187/cohort	160.7 (159.9–161.5)	158.5 (157.9–159.1)	–	9.6	11.9
2011 (26)	Brazil	16/cohort	164 (159–169) 0.07** (–0.43–0.12)	0.52** (0.12–0.92)	–	9.2	11.7
2012 (27)	Israel	73/retrospective	163.2 (161.9–164.5)	161.7 (160.4–163)	–	9.5	12.4
2018 (11)	Finland	30/case–control cohort	167.2 (164.8–169.6)	166.5	164.5 (162.7-166.3)	–	11.5
2019 (12)	Brazil	34/cross-sectional	162.8 (160.3–165,3)	–	–	–	11.1
Our report	Spain	79/cohort	164.6 (163.3–165.9) 0.58** (0.37–0.80)	163.6 (162.4–164.8) 0.40** (0.20–0.60)	–	9.7	11.6

*Mean (95% CI when possible).

**SDS.

The mean total growth from diagnosis until adult height was 32.95 cm. Otherwise, the total growth since Tanner stage 2 (B2) until adult height was 19.39 cm.

We built a correlation matrix with the different quantitative variables analyzed in this study. The initial univariate analysis showed that adult height was significantly correlated with target height (cm and SDS), birth weight (SDS), birth length (SDS), height at diagnosis (cm and SDS), predicted adult height (cm and SDS), and basal 17-OH-P (ng/ml) at diagnosis. Afterwards, several multiple linear regression models were designed using adult height as the dependent variable.

The best fit model had an *R* ^2^ of 68% after cross-validation (see **Table 4**). This model applied the following formula:

Adult height (cm) = 63.15 + target height (cm) × 0.23 + birth weight (SDS) × 0.63 + adult height prediction, taking in to account BA at diagnostic (cm) × 0.37 + height at diagnostic (SDS) ×2.28.

**Table 4:** Predictive models of adult height at diagnosis. Leave-one-out cross-validation of the models.

		Coef.	(95% CI)	*p*-value
**Model 1**	Constant	63.15	(31.82–94.47)	
	TH (cm)	0.233	(0.041–0.425)	0.018
	BW (SDS)	0.632	(0.014–1.250)	0.045
	PAH (cm)	0.368	(0.161–0.575)	0.001
	HD (SDS)	2.277	(0.969–3.585)	0.001
**Model 2**	Constant	85.71	(57.04–114.4)	
	TH (cm)	0.448	(0.270–0.625)	<0.001
	BL (SDS)	0.936	(0.142–1.730)	0.022
	HD (SDS)	3.370	(2.358–4.383)	<0.001
	**Absolute error**			
	**Mean ± SD**	**Range**	**Cross-validation *R* ^2^**	**Adjusted *R* ^2^**
**Model 1**	2.6 ± 1.6	(−6.5–6.7)	68%	71%
**Model 2**	2.8 ± 1.9	(−6.4–7.6)	63%	67%

TH, target height; BW, birth weight; PAH, predicted adult height at diagnosis; HD, height at diagnosis; BL, birth length.

This model displayed a 95% confidence interval for the prediction of ±2.6 cm.

We also evaluated different models without including the variable predicted adult height depending on BA at diagnosis. Thus, the best model achieved an *R* ^2^ after cross-validation of 63% with the following formula (see **Table 4**):

Adult height (cm) = 85.71 + target height (cm) × 0.45 + birth lenght (SDS) × 0.94 + height diagnosis (SDS) × 3.37.

This model showed a 95% confidence interval for the prediction of ±2.8 cm.

The percentage of girls diagnosed as having IPA with obesity at diagnosis was 17.72% (*n* = 14). At the moment of adult height evaluation, this prevalence was 21.51% (*n* = 17). Moreover, the correlation of BMI *Z*-score at diagnosis and at adult height evaluation was 0.71 (*p* < 0.001). Besides, 64.28% of obese girls at IPA diagnosis continued to be obese at adult height, while 35.72% decreased their BMI below 2 SDS. In addition, 13.85% of girls with normal weight at diagnosis were obese when they reached adult height.

## Discussion

In this study, we analyze data drawn from a cohort of girls with IPA evaluated periodically from diagnosis until adult height, with a mean total follow-up of 6.60 years. To our knowledge, no previous reports have designed a predictive model for adult height in female patients with this diagnosis.

We found that girls with IPA do not experience a loss in potential adult height, as adult height did not differ significantly from target height. In addition, the adult height SDS was within normal values according to Spanish references. Despite differences in inclusion criteria and protocol design, our data are consistent with previous reports (11, 12, 24–27). Ibañez et al. (25) studied the largest cohort of girls assembled for this purpose (*n* = 187); however, a high prevalence of SGA (birth weight < −2 SDS) was reported among the girls in the cohort (26.7%). This high percentage of SGA is only comparable to that reported by de Ferran et al. (26), who found a prevalence of 21.3%. Otherwise, only one report has prospectively included a control group for comparison with girls with IPA (11). This rigorously designed research had only 3.8% SGA, similar to our cohort.

Although the adult height of these girls is comparable to their target height, we found a decrease of height SDS at adult height with respect to height at diagnosis. This decline over time has been observed by other authors (10, 11) and is likely related to several factors such as advanced bone age at diagnosis, early puberty, as well as a decrease in total pubertal growth.

Despite the fact that adult height was not affected across our cohort, 17.7% of the girls had a difference of more than 1 SDS between their adult height and target height. So, how could we identify these cases? As in other endocrine disorders, BA at diagnosis has also been used to predict final adult height in girls with PA (28). However, the evaluation of BA presents several drawbacks. First, the Greulich-Pyle method is the most widespread procedure to calculate BA, and it is based on radiographs from upper-middle-class children recruited in the USA between 1931 and 1942. In addition, this evaluation procedure shows a relevant inter-observer variation. Otherwise, the Bayley–Pinneau method is the most commonly used system for predicting AH based on BA; this model has a 95% confidence interval of about 6 cm below to 6 cm above the predicted value. Finally, another drawback of BA assessment is the absence of unanimity when choosing between average and advanced options in the Bayley–Pinneau height prediction table (29). In order to improve the prediction of adult height provided by BA assessment, several studies have suggested different predictive models in central precocious puberty (14–16).

We observed a significant positive correlation (*r* = 0.66, *p* < 0.001) between adult height prediction at diagnosis by BA and adult height in IPA girls. Moreover, the mean difference between adult height and adult height predicted at diagnosis by the Bayley–Pinneau average age method was 1.71 cm with a 95% CI of 0.59 to 2.83 cm. This means that the adult height prediction is not overestimated. As previously remarked, however, the accuracy of this prediction depends substantially on clinician expertise (29).

The best model for adult height prediction shows an adjusted coefficient of determination (*R* ^2^) of 71% and includes target height, birth weight, height at diagnosis, and predicted adult height taking into account BA at diagnosis. In addition, we have achieved a 95% confidence interval of 2.6 cm below to 2.6 cm above the predicted value. After removing the variable of predicted adult height taking into account BA at diagnosis from the original formula, the adjusted coefficient of determination diminishes to 67% and the new 95% confidence interval is ±2.8 cm. With this second model, we can avoid the interpretation of BA by less experienced clinicians. Taking into account all the above, we think that models for predicting adult height in girls with IPA may be useful in detecting cases in which the predicted adult height is far from the target height. Therefore, we can schedule more personalized clinical follow-up.

As reflected in **Table 3**, our cohort has a mean age of onset of puberty and menarche similar to that reported by previous publications of girls with premature adrenarche (11, 12, 24–27). In 2008, Marco-Hernández et al. (30) published data from a Spanish cohort made up of 266 healthy Caucasian girls from the general population in which the mean age of breast development (B2) was 10.72 years and the mean menarcheal age was 12.43 years. Subsequently, García-Cuartero et al. (31) in 2010 showed data from a cohort of 301 healthy Caucasian children (195 girls) from the general population. In this study, the mean age of breast development (B2) was 10.1 years and the mean menarcheal age was 12 years. Although the study period is different, these data seem to indicate that IPA girls of our cohort present a modest pubertal advance with respect to healthy Spanish girls.

Another concern related to girls with IPA is the potential for an increased risk of obesity and metabolic complications (12, 13). With this cohort, we have been able to analyze the evolution of BMI from diagnosis to adult height. At diagnosis, 17.72% of girls with IPA were obese as compared to 21.50% at adult height. The percentage of obese girls at diagnosis is slightly higher than the obesity rate among Spanish girls in the same age range, i.e., 13.9% (32). Conversely, the percentage of obesity in adolescents with a history of IPA upon reaching adult height significantly exceeds the prevalence for Spanish girls between 13 and 17 years of age, which is 8% (33). Therefore, premature adrenarche increases the risk of obesity throughout pubertal development, and close monitoring of these girls is mandatory for weight control and to avoid possible metabolic complications.

Although all the girls included in this study underwent an ACTH stimulation test, it should be noted that this is a research project. Our intention was to reliably rule out congenital adrenal hyperplasia by performing this test and, in another way, to provide a cohort that was as homogeneous as possible in terms of performing the same diagnostic tests. In addition, we wanted to analyze the possible relationship between adult height and basal and stimulated 17-OH-progesterone values. Nevertheless, in the routine clinical practice, the evaluation of a girl with premature adrenarche must be individualized. Thus, the first step of biochemical tests to rule out congenital adrenal hyperplasia could include the determination of DHEA-S, testosterone, androstenedione, and basal 17-OH-progesterone in early morning (5, 34).

The main strength of our research is that it draws from a cohort evaluated every 6 months until adult height, with a mean follow-up of 6.60 years. Nevertheless, our study has certain limitations. The primary limitation is the absence of a control group. In our view, this limitation is more relevant in studies that compare pubertal development rather than adult height. Adult height can be compared with target height, and adult height SDS can be calculated according to population reference values. Another limitation is that our project was circumscribed to the catchment area of our hospital. It would be interesting to extend the study to other areas of Spain as well as to collaborate with groups from other countries in order to validate this initial model. It should be noted that we performed an internal validation of the models by the LOOCV method (23). Thus, the difference between Adjusted *R* ^2^ and Cross-validation *R* ^2^ was less than 5% in both models (**Table 4**), which constitutes a good outcome.

## Conclusions

IPA does not adversely affect adult height in girls. In some cases, however, adult height differs significantly from target height. Therefore, the implementation of predictive models of adult height on diagnosis of IPA could be useful for closer monitoring of selected girls.

In addition, due to the increased risk of obesity observed in our cohort at the end of puberty, it would be worthwhile to establish follow-up programs to monitor excess weight and prevent metabolic complications.

## Data Availability Statement

The original contributions presented in the study are included in the article/supplementary material. Further inquiries can be directed to the corresponding author.

## Ethics Statement

The studies involving human participants were reviewed and approved by the Research Ethics Committee of the University Hospital Fundación Jiménez Díaz. Written informed consent to participate in this study was provided by the participants’ legal guardian/next of kin.

## Author Contributions

LS-G contributed to the study conception and design. MS-C, AP-A, NC-R, and TG-P contributed to data collection. Preparation and analysis of materials were performed by FM-M and LS-G. IM-F helped with statistical analysis. The first draft of the manuscript was written by FM-M and LS-G, and all authors commented on pre-submission drafts. All authors contributed to the article and approved the submitted version.

## Funding

This research was carried out thanks to the support of a grant (nº PIC025-20) of the Familia Alonso Foundation.

## Conflict of Interest

The authors declare that the research was conducted in the absence of any commercial or financial relationships that could be construed as a potential conflict of interest.

## Publisher’s Note

All claims expressed in this article are solely those of the authors and do not necessarily represent those of their affiliated organizations, or those of the publisher, the editors and the reviewers. Any product that may be evaluated in this article, or claim that may be made by its manufacturer, is not guaranteed or endorsed by the publisher.
